# *In vitro* and *in vivo* performance of epinastine hydrochloride-releasing contact lenses

**DOI:** 10.1371/journal.pone.0210362

**Published:** 2019-01-30

**Authors:** Takahiro Minami, Waka Ishida, Tatsuma Kishimoto, Isana Nakajima, Shiori Hino, Ritsuko Arai, Toru Matsunaga, Atsuki Fukushima, Satoru Yamagami

**Affiliations:** 1 Department of Ophthalmology, The University of Tokyo, Bunkyo, Tokyo, Japan; 2 Department of Ophthalmology, Kochi Medical School, Nankoku, Kochi, Japan; 3 SEED CO., LTD., Bunkyo, Tokyo, Japan; 4 Department of Ophthalmology, Nihon University, Itabashi, Tokyo, Japan; University of Alabama at Birmingham, UNITED STATES

## Abstract

A number of drug-releasing contact lenses are currently being studied to address issues inherent in eye drops as a drug delivery method. In this study, we developed epinastine hydrochloride-releasing daily soft contact lenses for treatment of allergic conjunctivitis and examined their *in vitro* and *in vivo* performance. Preformed soft contact lenses with/without ionic functional groups were soaked in a solution of epinastine hydrochloride in phosphate-buffered saline to prepare epinastine hydrochloride-releasing soft contact lenses. Among these contact lenses with different ionicities, anionic lenses demonstrated the maximum, relatively linear epinastine hydrochloride release, *in vitro*. The amount of epinastine hydrochloride release was directly proportional to the concentration of the epinastine hydrochloride solution used to prepare the contact lens. The epinastine hydrochloride-releasing anionic soft contact lens also demonstrated prolonged drug release and significantly greater efficacy compared with epinastine hydrochloride eye drops 12 h after treatment, *in vivo*. Further studies are required to determine the appropriate amount of epinastine hydrochloride to be contained in the anionic soft contact lenses.

## Introduction

Eye drops are the most common form of ocular drug delivery, accounting for 90% of all the ophthalmic formulations [1]. The simple approach to the target organs, the eye and its adnexa, is generally supported by doctors and patients; however, ocular drug delivery by eye drops has several shortcomings, including low bioavailability, uncontrolled dispersion of the majority of the medication, unintended systemic or local side effects, and insufficient patient adherence [2].

To address these issues, a number of drug-releasing contact lenses have been studied [3]. Sustained drug delivery by contact lenses is considered to potentially overcome the above shortcomings of eye drops. Among several methods proposed so far to produce drug-releasing contact lenses, the simplest and most cost effective is to soak preformed contact lenses in a drug solution [3]. However, drug-releasing contact lenses made by the conventional soaking method have the following important limitations: relatively small drug loading ability and initial burst release of the drug [3]. Previous reports have shown that the soaking method can be modified for better drug delivery performance by incorporating ionic monomers into the contact lens polymer, which electrostatically interact with the drugs [3–5].

The purpose of this study was to examine the performance of epinastine hydrochloride (EH)-releasing daily disposable soft contact lenses (SCLs) made by the soaking method utilizing the ion ligand mechanism. EH, an effective selective histamine H1 receptor antagonist, is used as an anti-allergic drug to treat allergic conditions including allergic conjunctivitis [6]. Allergic conjunctivitis is a highly prevalent condition that has negative impacts on quality of life and the economy [7,8]. Considering that allergic conjunctivitis can be aggravated by contact lens use, and that patients with allergic conjunctivitis are usually advised to avoid using contact lenses, contact lenses that can prevent or treat allergic conjunctivitis by eluting anti-histamine drugs will be paradigm shifting in the management of allergic conjunctivitis and contact lens use [9]. There are only a few reports on contact lenses for anti-allergic drug delivery, and to the best of our knowledge, no report on EH-releasing contact lenses has been published [10,11]. In this study, we selected a potentially appropriate material for EH-releasing SCLs (EH-SCLs) from multiple newly synthesized candidates and evaluated the *in vivo* performance using the guinea pig allergic conjunctivitis model.

## Materials and methods

### Materials

The main non-ionic monomeric component, N-(2-hydroxypropyl)methacrylamide (HPMA), was obtained from Mitsubishi Chemical Corporation (Tokyo, Japan). Other non-ionic monomeric components, N-vinyl-2-pyrrolidone (NVP) and 1,4-cyclohexanedimethanol monoacrylate (CHDMMA), were obtained from Nippon Shokubai Co., Ltd. (Osaka, Japan) and Nihon Kasei Co., Ltd. (Tokyo, Japan), respectively. An anionic monomeric component, mono-2-(methacryloyloxy)ethyl succinate (HO-MS), was obtained from Kyoeisha Chemical Co., Ltd. (Osaka, Japan). A cationic monomeric component, [3-(methacrylamido)propyl]trimethylammonium chloride (MAPTAC), was obtained from MCC Unitech Co., Ltd. (Aomori, Japan). A crosslinking agent, polyethylene glycol 200 diacrylate (A-200), was obtained from Shin-Nakamura Chemical Co., Ltd. (Wakayama, Japan). A polymerization initiator, 2,2'-azobis(isobutyronitrile) (AIBN), histamine, and sodium sulfate (Na_2_SO_4_) were obtained from Fujifilm Wako Pure Chemical Corporation (Osaka, Japan). EH was obtained from Tokyo Kasei Kogyo Co., Ltd. (Tokyo, Japan). Evans blue dye (EB), and acetone were obtained from Nacalai Tesque, Inc. (Kyoto, Japan). Phosphate-buffered saline without divalent cations (PBS) was obtained from Fisher Scientific L.L.C. (Waltham, Massachusetts). All the reagents were used as received.

### Fabrication of SCLs with different ionicities

The monomers and the crosslinking agent were mixed according to the 5 composition ratios shown in Table 1. After 5000 ppm of AIBN was added to the liquid monomer mixtures to initiate polymerization, the mixtures were stirred for 2 h. The mixtures were then poured into uniform polypropylene custom-made molds for guinea pig contact lenses, and heated in the range of 30–90°C for 16 h to form 5 kinds of SCLs. The lenses were cooled to room temperature, removed from the molds, and soaked in PBS for more than 4 h to fully rehydrate. Following this, the lenses were submerged in 4 mL of PBS and autoclaved at 121°C for 30 min. The 5 kinds of SCLs which do not contain EH were named PBS-SCLs (A), (B), (C), (D), and (E). After the water on the lens surface was removed, each PBS-SCL was soaked in 2 mL of a filtered 0.05% (w/v) solution of EH in PBS for 24 h. These 5 kinds of epinastine hydrochloride-releasing soft contact lenses were named EH-SCLs (A), (B), (C), (D), and (E).

**Table 1:** Monomeric composition of the soft contact lenses with different ionicities.

EH-SCL	Ionicity	Monomeric composition (mol/100g)					
		HPMA	NVP	CHDMMA	HO-MS	MAPTAC	A-200
(A)	Anionic	0.72	-	0.08	0.08	-	0.0088
(B)	Cationic	0.69	-	0.08	-	0.11	0.0088
(C)	Bi-ionic	0.56	-	0.08	0.12	0.12	0.0088
(D)	Non-ionic	0.12	0.6	0.08	-	-	0.0080
(E)	Non-ionic	0.72	-	0.08	-	-	0.0080

Five kinds of soft contact lenses were fabricated with different monomeric compositions. To initiate polymerization, 5000 ppm of AIBN was added to each mixture of monomers.

EH-SCL: epinastine hydrochloride-releasing soft contact lens.

Anionic: containing anionic monomers and no cationic monomers.

Cationic: containing cationic monomers and no anionic monomers.

Bi-ionic: containing both anionic and cationic monomers.

Non-ionic: containing neither anionic nor cationic monomers.

HPMA: methacrylic acid, monoester with propane-1,2-diol, a non-ionic monomeric component.

NVP: N-vinyl-2-pyrrolidone, a non-ionic, hydroscopic monomeric component.

CHDMMA: 1,4-cyclohexanedimethanol monoacrylate, a non-ionic monomeric component.

HO-MS: mono-2-(methacryloyloxy)ethyl succinate, an anionic monomeric component.

MAPTAC: [3-(Methacrylamido)propyl]trimethylammonium chloride, a cationic monomeric component.

A-200: polyethylene glycol 200 diacrylate, a crosslinking agent.

### Water content of EH-SCLs

The weights of 3 SCLs of each kind were measured both before and after the PBS rehydration process. The water content of an EH-SCL was calculated by dividing the SCL weight differential by the SCL weight after rehydration.

### EH release profiles of different EH-SCLs

Three EH-SCLs of each kind were used in this study. Each EH-SCL was soaked in 250 μL of PBS, and 30 min later, the used PBS was collected as a sample and the EH-SCL was soaked in 250 μL of new PBS. In the same manner, PBS was replaced at 1, 2, 4, 6, 8, and 12 h after the initial soaking period started. At 36 h, the EH-SCL was soaked in 3 mL of new PBS for an additional 24 h in order to extract as much residual EH as possible.

The concentration of EH in each PBS sample was measured using high-performance liquid chromatography (Waters Corporation, Milford, MA) under the conditions shown in Table 2. This method was developed and optimized for this study, referring to the Japanese Pharmacopoeia and several other publicly available methods. The cumulative sum of the released EH during each soaking period was calculated from the measured concentration. The temporal cumulative sum of the released EH was divided by the total amount of EH released in 60 h, to evaluate the temporal change in the ratios of released EH.

**Table 2:** High performance liquid chromatography conditions.

Column	ACE 100 Å 5 μm C18 150 mm x 4.6 mm
Mobile phase	CH_3_CN/H_3_PO_4_(Na) = 30/70
Flow rate	1.0 mL/min
Detection	UV 220 nm

### EH release profiles with different EH concentrations

Each PBS-SCL (A) was soaked for more than 24 h in 2 mL of EH solution with 5 different concentrations: 0.003, 0.005, 0.008, 0.01, and 0.03% (w/v) (n = 3 for each concentration). As described above, the temporal cumulative sum of EH release from the 5 kinds of EH-SCLs with different EH concentrations were evaluated for 12 h.

### *In vivo* performance of the EH-SCL

This study was approved by the Animal Care Committee of Kochi Medical School (K-00118), and the procedures were performed according to the guidelines of the Association for Research in Vision and Ophthalmology *Statement for the use of Animals in Ophthalmic and Vision Research*. *In vivo* performance analysis of the EH-SCL was performed based on the previously reported animal model of allergic conjunctivitis [12]. In this animal model, the immediate phase of allergic conjunctivitis is induced by histamine eye drops, and the magnitude of the allergic reaction is measured by the extravasated EB, which was injected intravenously before the histamine challenge. Male Hartley guinea pigs aged 4–6 weeks were obtained from Japan SLC, Inc. (Shizuoka, Japan).

**Fig 1:**
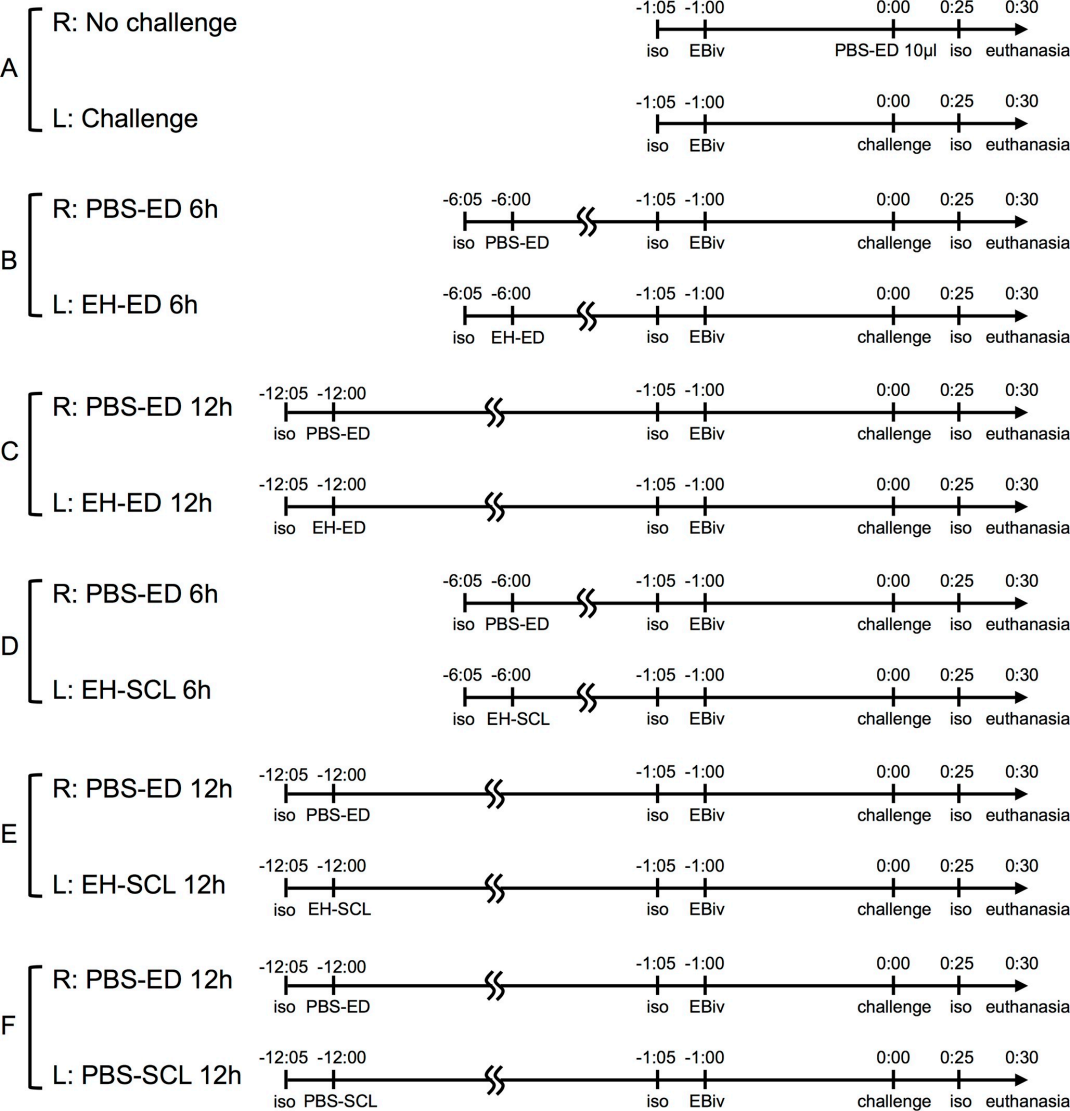
Schedule of the *in vivo* study. Scheduled procedures were undertaken in the 5 groups, A-E, of 6 guinea pigs each, and a group of 3 guinea pigs, F. iso: anesthesia by isoflurane inhalation, EBiv: intravenous injection of Evans blue, PBS-ED: instillation of eye drops of 50 μL of phosphate buffered saline, EH-ED: instillation of eye drops of 50 μL of a 0.05% (w/v) solution of epinastine hydrochloride in phosphate buffered saline. PBS-SCL: insertion of a soft contact lens containing no epinastine hydrochloride, EH-SCL: insertion of a soft contact lens containing epinastine hydrochloride, challenge: instillation of eye drops of 10 μL of a 1% (w/v) solution of histamine in phosphate-buffered saline (soft contact lenses were removed just before the challenge), euthanasia: decapitation under deep anesthesia with isoflurane inhalation.

We prepared 30 guinea pigs, and divided them into 5 groups (A, B, C, D, and E), 6 in each group. The different set of procedures described below were performed in each group according to the schedule shown in Fig 1. Anesthesia was induced using isoflurane inhalation. The body weight was measured, and 1.5 mL/kg of a 0.5% (w/v) solution of EB in PBS was injected into the veins of the auricle under temporary anesthesia. Histamine challenge was performed by pipetting 10 μL of a 1% (w/v) solution of histamine in PBS onto the surface of the eye. Thirty minutes after the challenge, decapitation was executed under deep anesthesia and the eyes and conjunctivae were harvested. The weight of the eye and conjunctiva together was measured for each tissue. Each tissue was placed in a 5-mL mixture of 0.5% Na_2_SO_4_ and acetone (3:7) for 48 h to extract EB. After centrifugation, the absorbance (620 nm) of the supernatant was determined using a spectrophotometer. The data are expressed as EB content (μg) per tissue weight (g). EH-SCLs used in this study were made by soaking PBS-SCLs (A) in 0.005% (w/v) EH solutions for more than 24 h.

In group A, no treatment was administered before the histamine challenge. The histamine challenge was performed only in the left eye, while 10 μL of PBS instead of a histamine solution was pipetted into the right eye. In groups B and C, 6 and 12 h before the histamine challenge, respectively, 50 μL of PBS (PBS-ED) was administered in the right eye, while 50 μL of a 0.05% (w/v) solution of EH in PBS (EH-ED) was administered in the left eye under temporary anesthesia. After each eye drop instillation, the eye was held open to retain the liquid inside the palpebral fissure for 1 min. The histamine challenge was performed in both eyes. In groups D and E, 6 and 12 h before the histamine challenge, respectively, PBS-ED was administered in the right eye, while an EH-SCL was inserted in the left eye, under temporary anesthesia. The EH-SCL in the left eye was removed just before the histamine challenge was performed in both eyes.

We prepared an additional group (F) of 3 guinea pigs. In group F, 12 h before the histamine challenge, PBS-ED was administered in the right eye, while a PBS-SCL was inserted in the left eye, under temporary anesthesia. The PBS-SCL in the left eye was removed just before the histamine challenge was performed in both eyes.

### EH extraction after *in vivo* use

On removal of the EH-SCLs in groups D and E, each EH-SCL was soaked in 3 mL of fresh PBS for 24 h to extract the residual EH. The samples of PBS used for EH extraction were collected, and the amount of EH was measured.

### Statistical analysis

The data were analyzed using JMP Pro Version 13.2.0 (64-bit) (SAS Institute Inc., Cary, NC, USA). The data are expressed as mean ± standard deviation. Statistical significance was determined by a two-tailed Student’s t-test, or where indicated, ANOVA and Tukey-Kramer test. *P* < 0.05 was considered to indicate statistical significance.

## Results

### Water content of EH-SCLs

**Fig 2:**
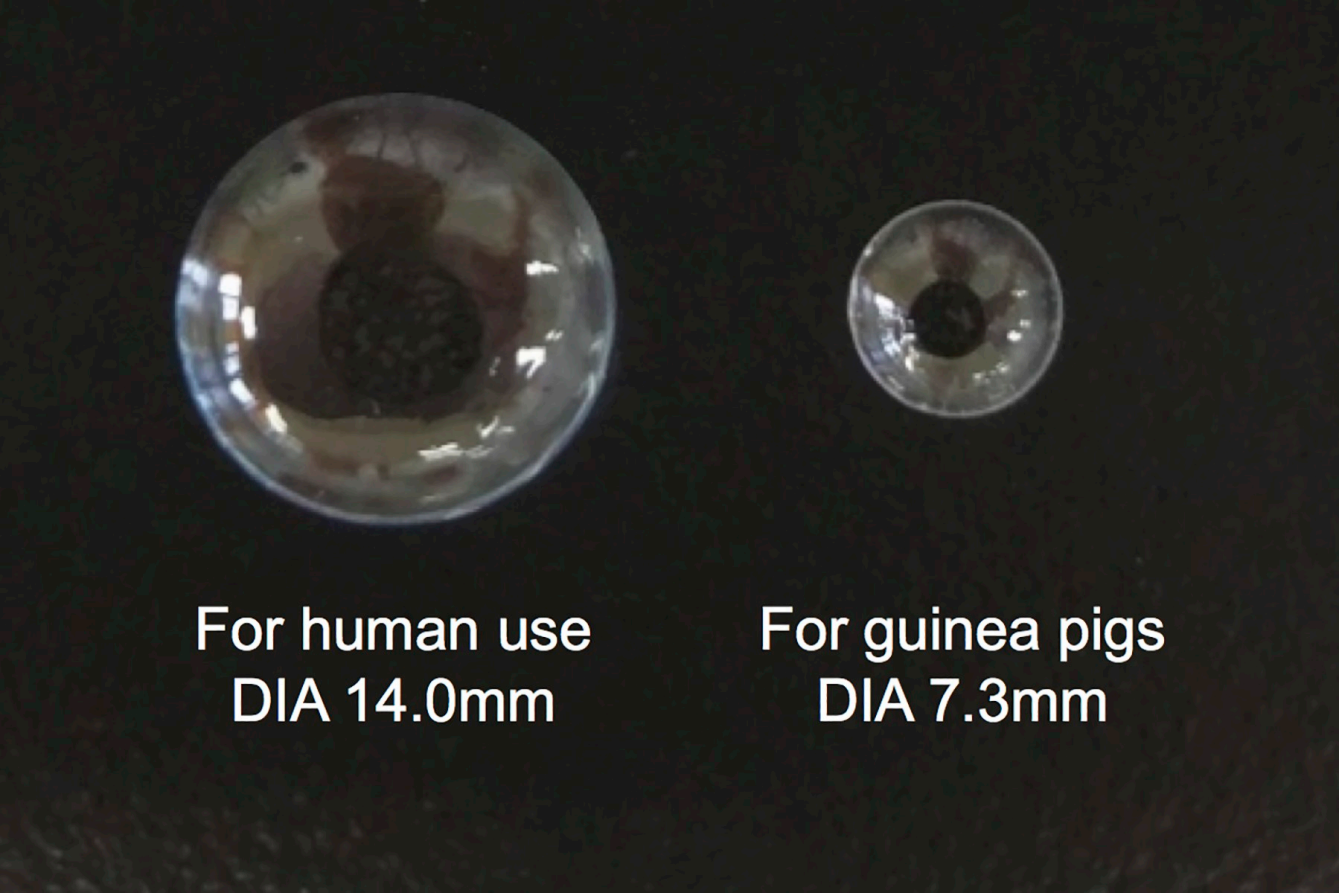
An epinastine hydrochloride-releasing soft contact lens (EH-SCL) for guinea pigs. An EH-SCL for guinea pigs is compared to an SCL for human use. DIA: diameter.

Five kinds of EH-SCLs for guinea pigs were fabricated (Fig 2, right); their water content are shown in Table 3. EH-SCLs (A), (B), (C), and (D) demonstrated similar water content (ranging between 54–59%). In contrast, the water content of EH-SCL (E) was 16.94 ± 0.96%.

**Table 3:** Water content.

EH-SCL	Water content (%)
(A)	58.4 ± 0.6
(B)	57.9 ± 1.0
(C)	58.2 ± 0.2
(D)	54.8 ± 1.0
(E)	16.9 ± 1.2

Water content of 5 kinds of soft contact lenses are shown. Data are means ± standard deviations (n = 3). EH-SCLs (A), (B), (C), (D), and (E): epinastine hydrochloride-releasing soft contact lenses with different ionicities.

### EH release profiles of different EH-SCLs

**Fig 3:**
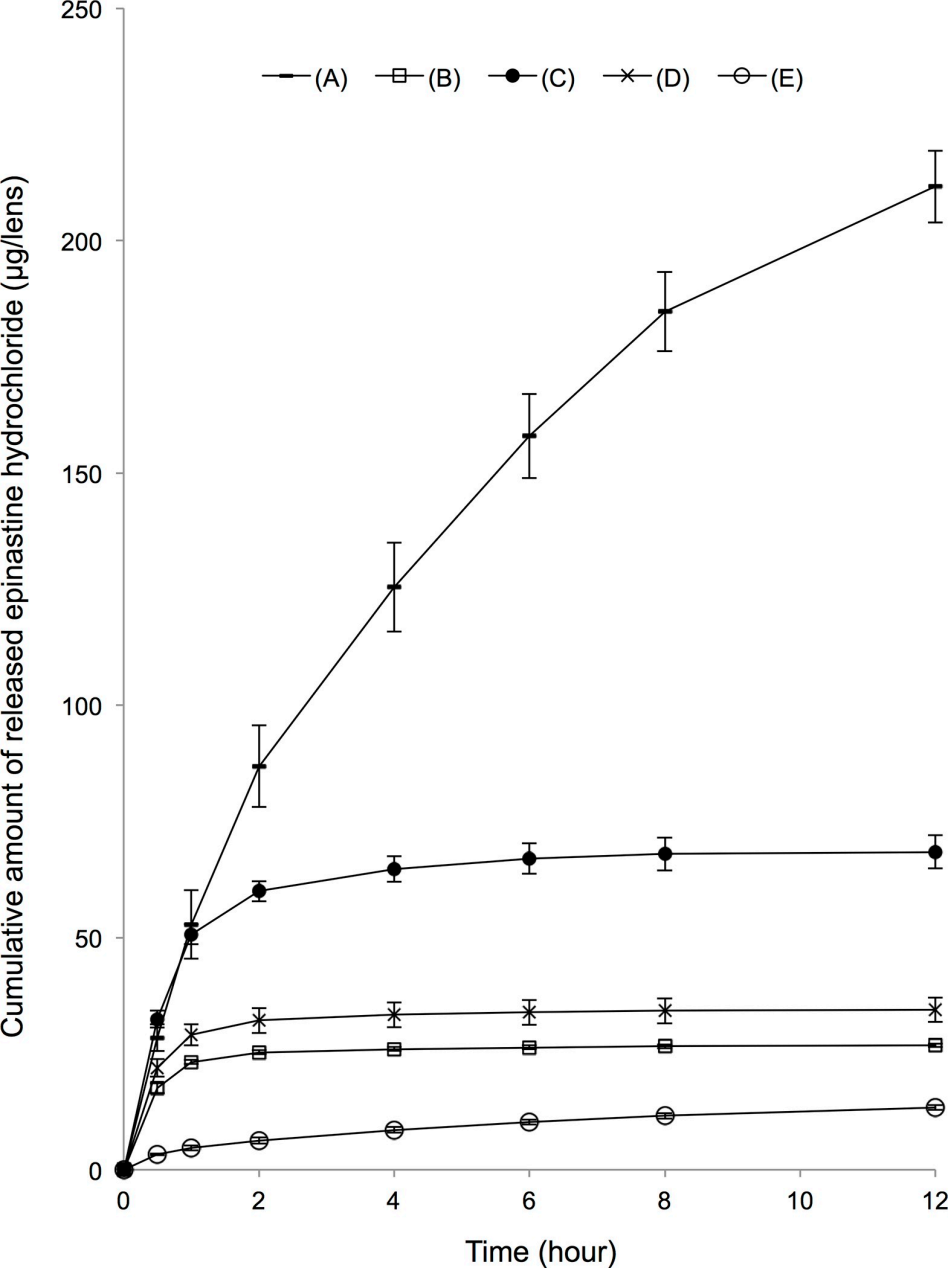
Epinastine hydrochloride (EH)-releasing profiles of the 5 kinds of soft contact lenses (EH-SCLs). The maximum EH release was demonstrated by EH-SCL (A) after 2 h. Data are expressed as mean ± standard deviation (n = 3).

**Fig 4:**
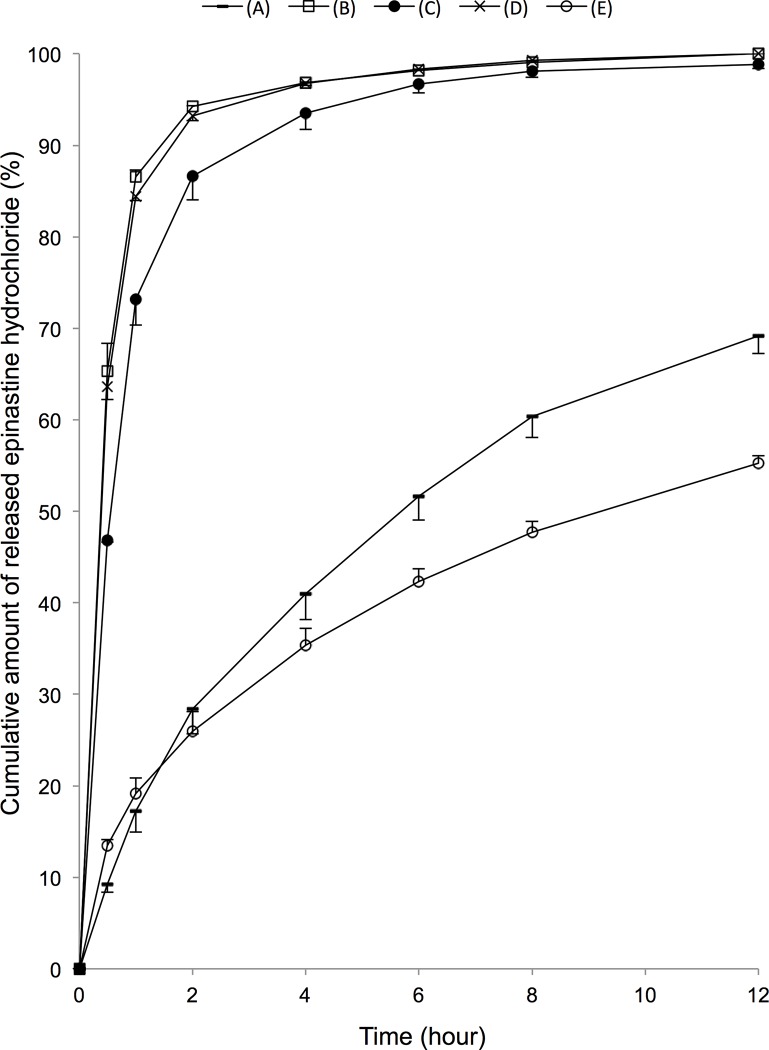
Temporal change in the ratios of cumulative amount of released epinastine hydrochloride (EH). Ratios of temporal cumulative amounts of EH released to the total amount released in 60 h are shown for the 5 kinds of EH-SCLs (n = 3). Relatively stable release profiles were demonstrated by EH-SCLs (E) and (A).

Temporal cumulative amount of EH released from different EH-SCLs are shown in Fig 3, and the ratio of the temporal cumulative amount of EH to the total amount of EH released in 60 h is shown in Fig 4. The raw data and the statistical information are presented in the supplementary data files (S1 Dataset and S1 Stats). EH-SCL (A) demonstrated the largest amount of EH release, 211.60 ± 7.70 μg/lens in 12 h, and 305.80 ± 3.84 μg/lens in 60 h. This was followed by EH-SCL (C), at 68.42 ± 3.61 μg/lens in 12 h, and 69.23 ± 3.84 μg/lens in 60 h. EH-SCLs (B) and (D) demonstrated relatively small amounts of EH release in 12 h, 26.81 ± 0.39 μg/lens and 34.44 ± ±2.68 μg/lens, respectively, and no more release later. EH-SCL (E) demonstrated the smallest amount of EH release, 13.40 ± 0.51 μg/lens in 12 h and 24.24 ± 0.58 μg/lens in 60 h.

EH-SCL (E) demonstrated the most linear EH release over time with the release ratio 42.3% in 6 h and 55.3% in 12 h, followed by the EH-SCL (A) with 42.3% in 6 h and 69.2% in 12 h. EH-SCLs (B), (C), and (D) demonstrated relatively steep initial burst release; the release ratios were more than 95% in 6 h. EH-SCLs made by soaking PBS-SCLs (A) in EH solutions were selected for the further consideration in this study, due to the relatively efficient and linear EH release.

### EH release profiles with different EH concentrations

**Fig 5:**
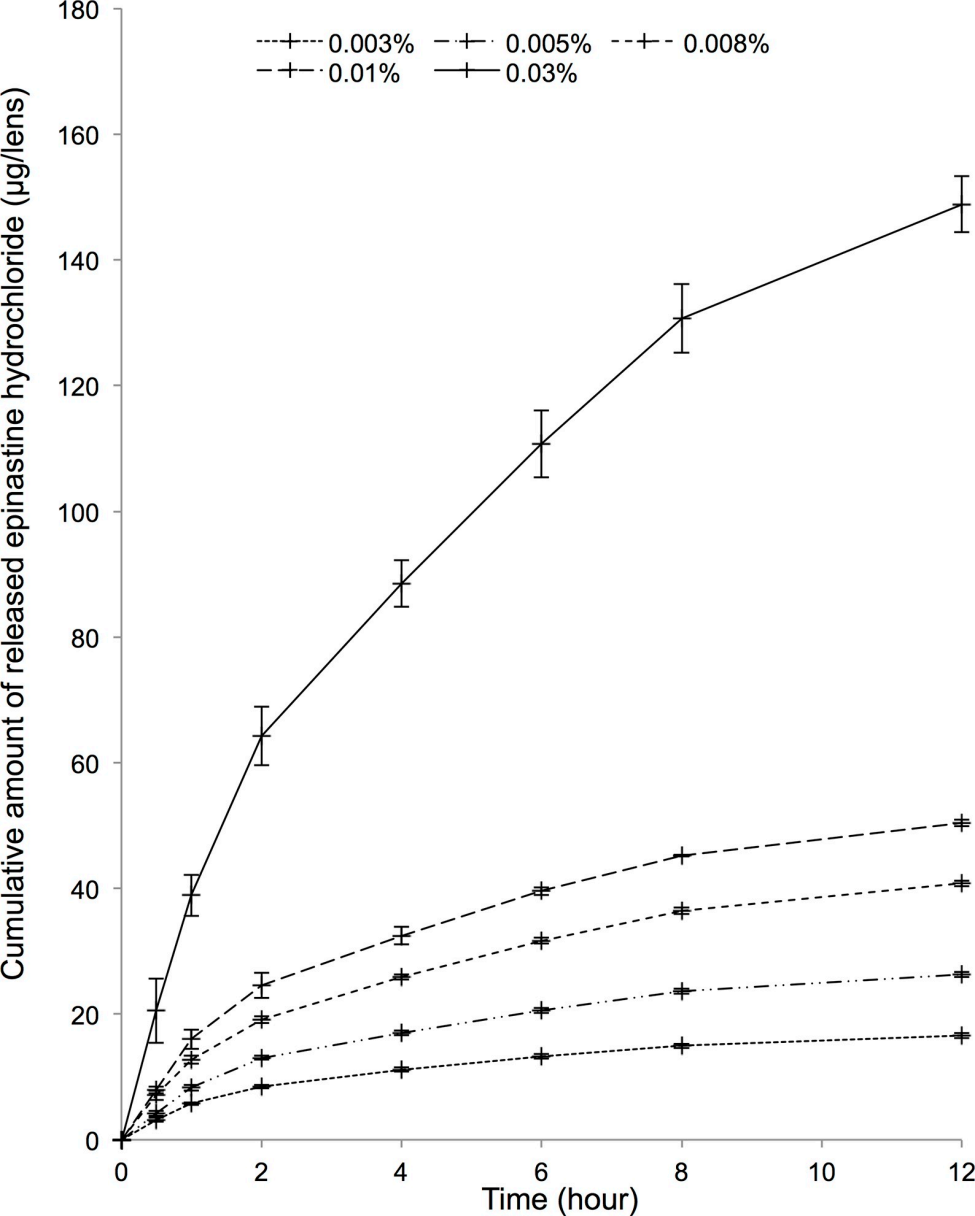
Epinastine hydrochloride (EH) releasing profiles with different EH concentrations. The amount of EH release increased as the concentration of the EH solution used to make the EH-releasing soft contact lenses increased.

**Fig 6:**
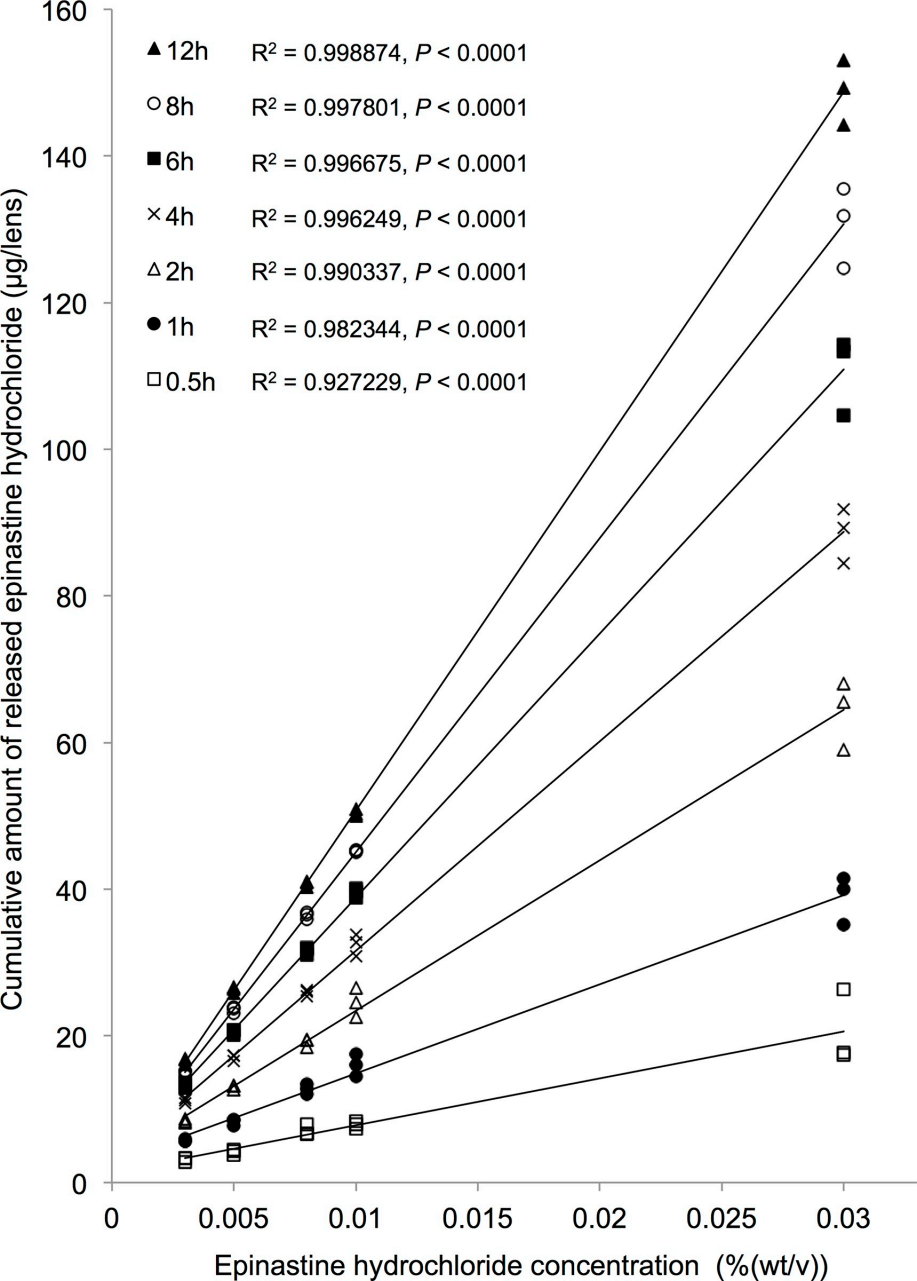
Correlations between epinastine hydrochloride (EH) concentrations and the release amounts. The EH-releasing soft contact lenses (EH-SCLs) demonstrated EH release directly proportional to the concentration of EH solutions used to make the EH-SCLs.

The EH release profiles of EH-SCLs made by soaking PBS-SCLs (A) in EH solutions with different concentrations are shown in Fig 5. The amount of EH release increased as the concentration of EH solution used to make the EH-SCL increased. The EH release was directly proportional to the EH concentrations at any period of time (Fig 6). This result indicated that the amount of EH released from an EH-SCL can be adjusted by the concentration of the EH solution used to make the EH-SCL, at least in the *in vitro* setting. For the *in vivo* study, we decided to use a 0.005% EH solution to prepare EH-SCLs which released approximately the same amount of EH in 12 h (26.29 ± 0.44 μg/lens) as 50 μL of 0.05% commercially available EH in PBS eye drops (25 μg of EH).

### *In vivo* performance of the EH-SCL

**Fig 7:**
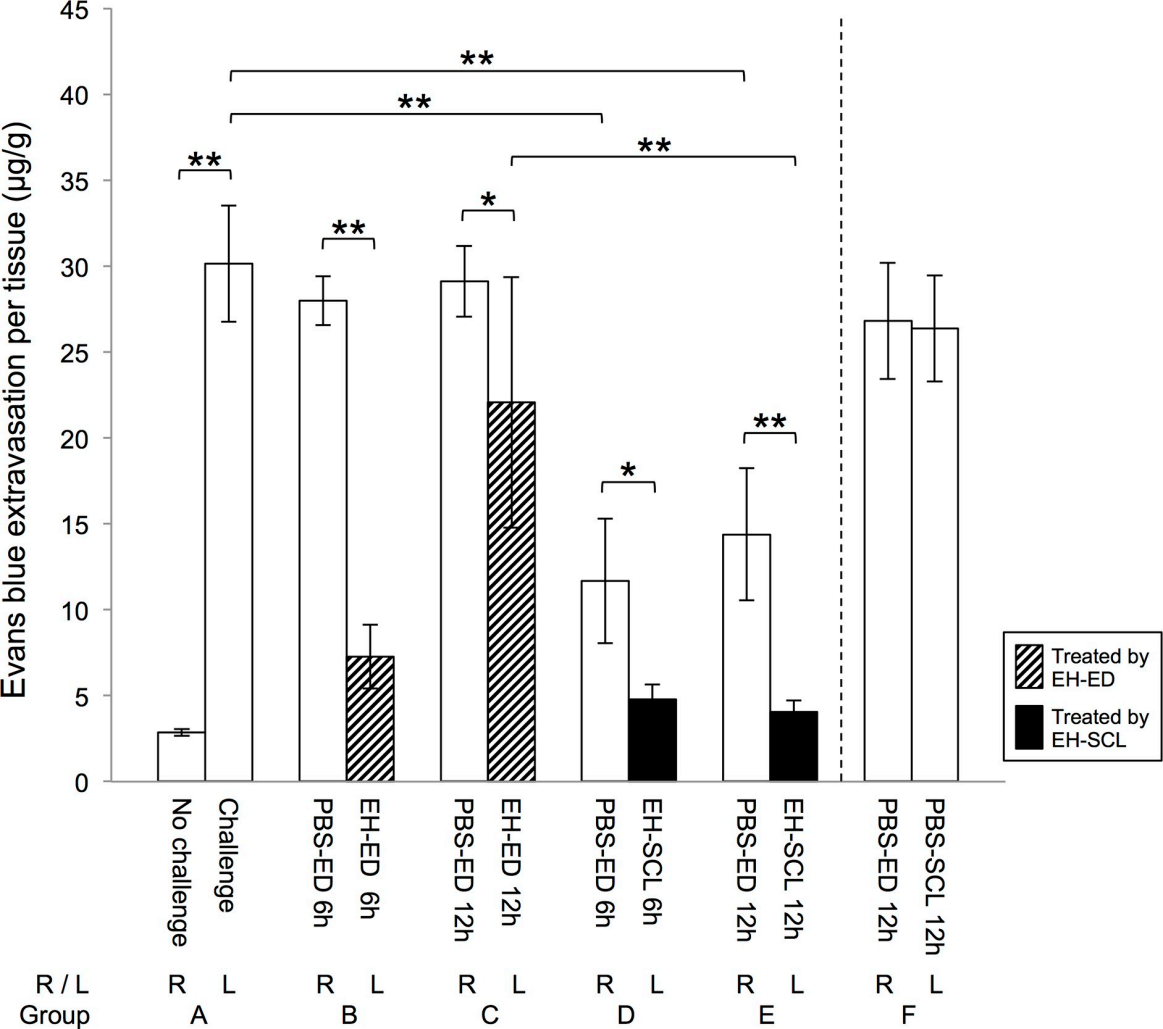
Evans blue (EB) extravasation per tissue. The extravasation of EB was significantly inhibited in the eyes in which epinastine hydrochloride (EH) was administered in the form of either eye drops or soft contact lens. Twelve hours after each treatment, EH-SCLs made with a 0.005% solution of EH inhibited EB extravasation significantly more compared with EH-EDs. Groups A to E, n = 6; for group F, n = 3. For groups A to E, the data were analyzed using one-way ANOVA and Tukey-Kramer test. * and ** indicate *P* < 0.05 and *P* < 0.01, respectively. PBS-ED: treated with phosphate-buffered saline eye drops, EH-ED: treated with epinastine hydrochloride eye drops, PBS-SCL: treated with a soft contact lens containing no epinastine hydrochloride, EH-SCL: treated with a soft contact lens made with a 0.005% solution of epinastine hydrochloride.

The results of the *in vivo* study using EH-SCL made by soaking PBS-SCL (A) in a 0.005% EH solution are shown in Fig 7. In group A, EB extravasated from the right eyes (no treatment, no challenge) and left eyes (no treatment, challenge) were 2.87 ± 0.192 μg/g and 30.13 ± 3.39 μg/g, respectively, with a significant difference (*P* < 0.0001). In groups B, C, D, and E, EB extravasated from the left eyes (EH-EDs or EH-SCLs for 6 or 12 h) were all significantly less than that from the right eyes (PBS-EDs for 6 or 12 h); *P* < 0.0001, *P* = 0.0137, *P* = 0.0173, and *P* = 0.0048 for groups B, C, D, and E, respectively. The raw data are presented in the supplementary data file (S1 Dataset). These results indicated that the immediate reaction to the histamine challenge induced extravasation of EB and EH inhibited the EB extravasation.

In group F, there was no significant difference in EB extravasation between the right (26.81 ±3.4 μg/g; PBS-EDs for 12 h) and left eyes (26.37 ± 3.10 μg/g; PBS-SCLs for 12 h) (*P* = 0.8746). There was also no significant difference in EB extravasation between the right and left eyes in group F and the left eyes in group A (no treatment) (*P* = 0.1924, and *P* = 0.1447, respectively). These results indicated that the soft contact lens by itself did not have an effect on EB extravasation in this animal model.

There was no significant difference in EB extravasation between the eyes treated with EH-EDs for 6 h (group B, left eyes) and those treated with EH-SCLs for 6 h (group D, left eyes) (*P* = 0.9398). On the other hand, EB extravasation from the eyes treated with EH-SCLs for 12 h (group C, left eyes) was significantly less than that from the eyes treated with EH-EDs for 12 h (group E, left eyes) (*P* < 0.0001).

Regarding the eyes treated with PBS-EDs for 6 h or 12 h (groups B, C, D, and E; right eyes), EB extravasated from the right eyes in groups B and C was not significantly different from each other (P = 0.9998), and also not significantly different from that extravasated from the left eyes in group A (no treatment) (*P* = 0.9756, and *P* = 0.9999, respectively); EB extravasated from the right eyes in groups D and E was not significantly different from each other (P = 0.9014), and less than that from the left eyes in group A (no treatment) (*P* < 0.0001 for both).

Comparing the eyes treated with EH for 6 h and 12 h, more EB extravasation was observed from the eyes treated with EB-EDs for 12 h (group C, left) than those treated with EB-EDs for 6 h (group B, left) (*P* < 0.0001). There was no difference in EB extravasation between the eyes treated with EB-SCLs for 6 h (group D, left) and those treated with EB-SCLs for 12 h (group E, left) (*P* = 1.0000).

### EH extraction after *in vivo* use

The amount of EH extracted from EH-SCLs in groups D and E after *in vivo* use were 12.39 ± 0.52 μg/lens and 3.89 ± 0.78 μg/lens, respectively.

## Discussion

The EH releasing contact lenses were fabricated and examined for the first time in this study. EH-SCLs (A), (B), and (C) contained anionic, cationic, and both kinds of monomers, respectively, and demonstrated high water content, around 58%. Although EH-SCL (D) didn’t contain ionic monomers, EH-SCL (D) had similar water content to EH-SCL (A), (B), and (C). The hydroscopic nature of NVP is considered to have contributed to the high water content of EH-SCL (D). On the other hand, another non-ionic material, EH-SCL (E), had low water content, around 17%. The less water-soluble property of the main components of EH-SCL (E), HPMA and CHDMMA, is considered to have caused the low water content. The amount of drug a contact lens can absorb and release is influenced by its water content [13]. Thus, since EH-SCLs (A)-(D) have similar water content, these lenses are suitable to examine the ionicity and the drug release profile. Additionally, EH-SCLs (A), (B), (C) with high water content (>50%) and the ionic properties would be categorized into group IV in the hydrogel grouping by the United States Food and Drug Administration; EH-SCL (D) into group II; EH-SCL (E) into group I.

Among the EH-SCLs with high water content, the amount of EH released in the *in vitro* study varied greatly. In 12 h, the EH-SCL (A) (anionic) released by far the largest amount of EH, followed by EH-SCL (C) (anionic and cationic), (D) (nonionic), and (B) (cationic), in that order. Regarding the ratio of temporal cumulative amount of EH released to the total amount in 60 h, EH-SCLs (B), (C), and (D) demonstrated steep initial burst, releasing more than 80% of EH in 2 h, and 100%, 98.8%, and 100% in 12 h, respectively. In contrast, EH-SCL (A) demonstrated a relatively linear release profile, where 69% of EH was released in 12 h. It is considered that electric interaction between EH, a cationic molecule with an amino group, and HO-MS, an anionic molecule with carbonyl groups, enabled EH-SCL (A) to absorb and gradually release a lot of EH, and that without the ionic interaction, EH-SCL (D) did not have the ability. On the other hand, electric repulsion between MAPTAC, a cationic monomer with a trimethylammonium group, and EH with the same cationic nature is considered to have inhibited EH-SCLs (B) and (C) from absorbing EH, and to have promoted the SCLs to release large proportions in a short period. These results are consistent with previous reports where drugs were efficiently absorbed and released over extended periods, due to the ionic interactions between drugs and polymers of opposite ionic natures [5,13,14]. The result of this study is also compatible with reports where the uptake and release amount of a cationic drug (Ciprofloxacin or Ketotifen) in different kinds of commercially available soft contact lenses were examined, and Etafilcon A, a group IV material, demonstrated larger uptake and release than Polymacon, a group I (low water, non-ionic) material, or Alphafilcon A, a group II (high water, non-ionic) material [10,15].

In the *in vivo* study, 6 hours after instillation of EH-EDs or application of EH-SCLs in the left eyes of groups B or D, there was no significant difference in EB extravasation in the two groups, while EB extravasation showed wide variation in the eyes treated by EH-EDs (the left eyes in group C), but was inhibited consistently in the eyes treated by EH-SCLs (group E, left) in 12 h. There was a significant difference between the eyes treated by EH-EDs and those treated by EH-SCLs. These results indicated that initially EH-EDs and EH-SCLs demonstrated similar effectiveness, but after 6 h, while the effectiveness of the EH eye drops waned, the EH-SCLs remained effective, releasing EH. Assuming the daily disposable use of these contact lenses, the EH-SCL displayed a competent drug delivery capability.

The difference in extracted EH from EH-SCLs used in the *in vivo* study for 6 and 12 h (12.39–3.89 = 8.50 μg/lens) can be interpreted as the amount of EH released in the eyes in the 6-to-12 h period, and is considered to have contributed to the continued inhibition of EB extravasation. This amount of EH, released in the *in vivo* study, was larger than that observed in the *in vitro* study in the same 6–12 h period (5.70 μg/lens). This may have been caused by the difference of environment of the EH-SCL between *in vitro* and *in vivo* studies. Though we aimed to design the EH-SCL to release 25 μg of EH in 12 h in the animal eye, the actual amount of released EH may have been larger.

As for the eyes treated with PBS eye drops, EB extravasation was significantly inhibited in the right eyes in groups D and E, but not in the right eyes in groups B and C. In a pharmacology review of EH by the US Food and Drug Administration, it was reported that after administration of repeated unilateral ocular doses of ^14^C-epinastine, low concentrations of radioactivity were measured in the tissues of the untreated eye, indicating that radioactivity entered the untreated eye from systemic circulation [16]. In our study, the right eyes in groups D and E were the untreated eyes, and it is considered that EH released from EH-SCLs entered the contralateral untreated eyes via the systemic circulation, inhibiting EB extravasation.

A considerable amount of the medication was dispersed upon release of the eyelids, though the eyes were held open for 1 min after the instillation of the eye drops under general anesthesia,. In humans, it is reported that only 1–7% of the medication in eye drops is absorbed [17]. Conversely, since it is unlikely that any EH released from an EH-SCL escaped absorption by the tissue, all the released EH is considered to have entered the animal body. Though the EH-SCL was made with an EH solution of 1/10 of the concentration of the EH eye drops for human use, the EH-SCL demonstrated significantly greater efficacy than did the EH eye drops at 12 h, and also demonstrated an unexpected effect on the untreated contralateral eye, which suggests that the amount of EH released from EH-SCL to the eye exceeded an appropriate therapeutic level. Considering the higher bioavailability of drugs delivered by a contact lens and the relatively consistent drug release over an extended period, it is opined that the total amount of drug contained in a contact lens should be kept lower than that conventionally present in eye drop formulations. Though this study demonstrated the feasibility of the one-day disposable EH-releasing contact lens, it is still not clear whether the primary contribution of the long-term *in vivo* effect was due to the extended release of EH or its high bioavailability. In order to optimize the dose of EH and the EH release profile of the lens material, further studies are needed.

As a different drug delivery method, instillation of eye drops over an SCL can be another option. In this method, the SCL may work as a reservoir that adsorbs and releases some of the drugs in the eye drops, as a container of the eye drop solution on the eye, or as a shield to prevent the eye drop from contacting the surface of the eye. In any case, considering the result of this study, due to the small amount of the eye drop retained on the eye after blinking, drug delivery by SCL is expected to demonstrate more potent *in vivo* effect. Moreover, the consistency of the effect of the eye drops over an SCL may be affected by the varied amount of tear fluid between the eye and the SCL.

There are some other clinically important issues that need to be addressed in follow-up studies, including optical property and shelf life. Contact lenses sometimes get colored when they absorb drugs, and drug release often accompanies change of contact lens shape [5,13]. In EH-SCLs, apparent discoloration or shape change were not observed, with the clear and colorless look and the stable fit on the guinea pig eyes maintained over 12 h. However, these optical properties must be precisely investigated. Regarding storage, contact lenses made by the soaking method are generally easy to store without major drug leaching at stable temperatures [3]. However, to account for extreme environmental conditions, *in vitro* and *in vivo* drug release profiles of EH-SCLs must be examined at different temperatures and storage periods.

## Conclusions

We created the first prototype of EH-SCLs with different ionicities by the soaking method utilizing ion ligand mechanism. Anionic EH-SCLs demonstrated the largest and relatively linear release of EH. The amount of EH released from the EH-SCL in the *in vitro* study was directly proportional to the concentration of the EH solution used to prepare the EH-SCL. The EH-SCLs also demonstrated prolonged drug release and significantly greater efficacy than did the EH eye drops over 12 h in the *in vivo* study. Further studies are needed to determine the appropriate amount of drug to be contained in the EH-SCL.

## Supporting information

S1 Dataset Original data used in this article are included. (XLSX)

S1 Stats Additional statistical data of the *in vivo* study are included. (XLSX)
